# “Border closure only increased precariousness”: a qualitative analysis of the effects of restrictive measures during the COVID-19 pandemic on Venezuelan’s health and human rights in South America

**DOI:** 10.1186/s12889-023-16726-0

**Published:** 2023-09-21

**Authors:** Teresita Rocha-Jimenez, Carla Olivari, Alejandra Martínez, Michael Knipper, Báltica Cabieses

**Affiliations:** 1 Society and Health Research Center, School of Psychology, Faculty of Social Sciences and Arts, Universidad Mayor, Santiago, Chile; 2 Millennium Nucleus On Sociomedicine, Santiago, Chile; 3 Center for Technology and Behavioral Health, Dartmouth College, Hanover, New Heaven USA; 4 Global Health, Migration and Medical Humanities, University of Giessen, Giessen, Germany; 5 Board of Lancet Migration Latin America, Lima, Peru; 6 Centre for Global Intercultural Health (CeSGI), Instituto de Ciencias e Innovación en Medicina (ICIM), Facultad de Medicina Clínica Alemana, Universidad del Desarrollo, Santiago, Chile

**Keywords:** Human rights, Health, Venezuelans, Chile’s northern border

## Abstract

**Background:**

In 2010, a political and social crisis pushed thousands of Venezuelans out of their country; today, seven million Venezuelans live abroad. In addition, during the COVID-19 pandemic, border closure increased and affected specific vulnerable migration flows, such as Venezuelans trying to migrate to Chile through the Northern borders. In this context, there is little evidence of migrants’ health status and needs, their access to health services, and other basic needs (e.g., housing) from a human rights perspective. Therefore, we qualitatively explored the effects of border closure due to the COVID-19 pandemic on Venezuelan migrants’ health and human rights, focusing on access to healthcare in the Northern Chilean border that adjoins Peru and Bolivia.

**Methods:**

Following a case-study qualitative design, we conducted an ethnography that included participatory observation of relevant sites (e.g., hospitals, main squares, migrant shelters) in Antofagasta, Iquique, and Arica and 30 in-depth interviews with actors in the health sector (*n* = 7), experts from the non-governmental sector (*n* = 16), and governmental actors (*n* = 7) in three large cities close to the Northern border.

**Results:**

We found four main dimensions: (i) border and migration processes, (ii) specific groups and intersectionality, (iii) barriers to healthcare services, and (iv) regional and local responses to the crisis during the COVID-19 pandemic. Programs characterized by the presence of healthcare providers in the field were essential to attend to migrants’ health needs at borders.

**Conclusions:**

Coordination between actors is crucial to implement regional protocols that respond to current migration phenomena and migrants’ health needs. Health policies using a human rights approach are urgently required to respond to migrants’ healthcare needs at borders in South America.

## Background

Globally, there are 272 million international migrants, representing almost 4% of the world’s population. In 2021, there were 21.3 million refugees, 4.6 million asylum seekers, and 51.3 million internally displaced people [1]. Migration and mobility have been associated with individuals’ health outcomes; some might be positive or negative [2, 3]. For example, studies around the world have documented that, in some cases, migration and mobility are essential factors of poverty alleviation [3, 4]. Eventually, this may translate into increased access to healthcare services and better health outcomes [2, 5]. However, migration and mobility, especially displacement, may also entail higher levels, social isolation, and barriers to health services due to discrimination, irregular migration status, and lack of health services’ intercultural competences [5, 6]. Eighteen percent of the population of concern to the United Nations High Commissioner for Refugees is in the Americas; this population includes Venezuelan refugees, asylum seekers, internally displaced people (IDPS), returned IDPs, and displaced abroad [1]. These numbers are likely to increase, given the ongoing natural disasters due to climate change, current wars, internal conflicts, and the implementation of restrictive migration policies [7–10].

In 2010, a political and social crisis pushed thousands of Venezuelans out of their country with seven million Venezuelans living abroad today. The exodus of Venezuelans, due to the political, economic, and social crisis, is considered the largest in Latin America in the last 50 years, which some classify as a “humanitarian emergency” [11, 12]. In Latin America, the main destination for Venezuelans is Colombia (2.5 M), followed by Peru (1.5 M), and Chile (450 thousand) [13]. In 2018, in an attempt to respond to the Venezuelan humanitarian crisis, the regional governments of Argentina, Brazil, Chile, Colombia, Ecuador, Mexico, Panamá, Paraguay, Peru, and Uruguay signed the Quito Agreement, an action plan to face the migratory and humanitarian crisis [11]. This agreement included efforts to adequately attend to the needs of the displaced Venezuelan citizens, especially the most vulnerable such as children, adolescents, older adults, people with disability, and with health conditions. Another essential factor of this agreement was guaranteeing Venezuelans’ human rights in the region [11] by implementing regional efforts to exchange information and articulate programs. However, once the COVID-19 pandemic arrived, some of these efforts were harder to implement, and each country faced specific challenges.

International borders are defined by the International Migration Organization as “politically defined boundaries separating territory or maritime zones between political entities and the areas where political entities exercise border governance measures on their territory or extraterritorially [14]. Such areas include border crossing points (airports, land border crossing points, ports), immigration and transit zones, the “no-man’s land” between crossing points of neighboring countries, as well as embassies and consulates (insofar as visa issuance is concerned)” [15]. Closure of an international border entails the restriction of international mobility by closing borders, controlling entrances, visa changes, and entry requisites [16]. Historically border controls and quarantines have often been used as public health measures to control infectious diseases [17, 18]. However, in our current globalized world, these measures are harder to implement, and they also entail economic, social, and humanitarian costs threatening individuals’ lives, health, and human rights [19, 20]. For this paper, we will focus on specific human rights in border contexts, which include the right to have information on migration processes, the right to family reunification, the right to access health services, the right to cultural identity, the right to a nationality, prohibition of deportation or if this happens, to understand the reasons, and ban on human trafficking [21]. There is evidence from before the COVID-19 pandemic, of how restrictive border measures affect circular migration flows such as everyday or seasonal workers and commuters and how restricting borders also increases the risks for those crossing through irregular points of entry by pushing migrants and asylum seekers to dangerous circumstances (e.g., dehydration, accidents, assaults, and death). Finally, restricting borders also affects family separation, trauma, and the overall wellbeing of those who migrate and their families [22, 23].

**Fig. 1:**
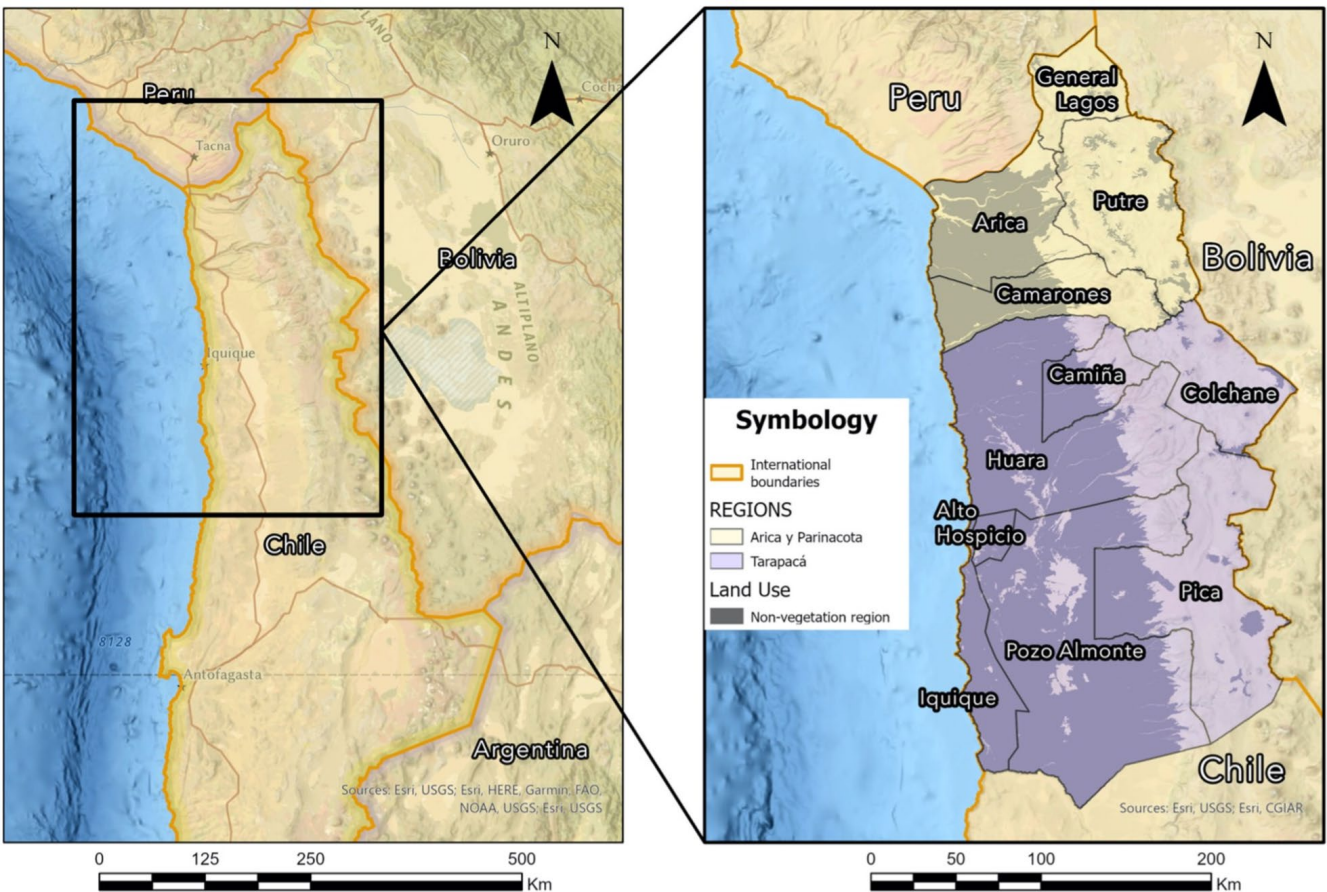
The Northern Chilean border

Chile closed its borders with Peru and Bolivia in March 2019; no one could cross through a formal land point of entry, including everyday commuters and seasonal workers [24]. Overall, the measures implemented in Chile to prevent the spread of COVID-19 were strict; and included mobility restrictions between neighborhoods and regions, curfews, the requirement of online permits to buy food, and the closure of all non-basic services [25]. For populations working in the informal economy, this entailed receiving less income or not receiving it at all [26]. Furthermore, thousands of seasonal migrants remained stranded in Chilean territory for months before they could return to their country of origin [24, 27]. Despite the efforts to prevent the entrance of people and, therefore, the spread of COVID-19, irregular border crossings considerably increased. Sixty-one percent of the total irregular crossings notified since 2010 were registered between January 2018-September 2020 [28]. The primary nationality crossing through the Northern Chilean borders was Venezuelan and more than 50% of such crossings were registered in the areas of Arica and Parinacota and Tarapacá (See Fig. 1) [25, 28]; Venezuelans represent the largest group of international migrants in Chile, constituting almost half a million people [29]. Border closures facilitated irregular migration and favored clandestine networks of smugglers and human traffickers, which again amplified the precariousness of migrants’ crossing conditions [19, 25, 30].

In this context, overall health services in Chile were critically affected by the urgent need to address COVID-19 cases. All the resources, including medical staff, were focused on attending to the most urgent cases. In the peak of the pandemic, Chile registered a case fatality rate of 2.7% (December 2020) [31], and in 2020 former president Sebastian Piñera reported that the healthcare capacity was close to its limit, given the critical respiratory cases [32]. Primary care and chronically ill patients were severely affected by the need to attend to COVID-19 cases, evidencing the Chilean health system’s weaknesses [33]. This circumstance was not unique to Chile and was seen during the COVID-19 pandemic in many other Latin American Countries [20]. Specific vulnerable groups were more affected by this reality, including migrants and especially those who had recently arrived [30]. Given the migration status of the recently arrived population and the fact that they were entering Chile during the sanitary emergency of COVID-19, there is little evidence of migrants’ health status and needs, their access to health services and other basic needs (e.g., housing, food, and education) [12, 30, 34]. Therefore, this paper aims to qualitatively explore the effects of border closure due to the COVID-19 pandemic on Venezuelan migrants’ health and human rights in Chile by focusing on understanding the process of migration and mobility at the borders, the access to health services, and the regional and local responses.

## Methods

### Study description

The present study is part of a multi-site project that aims to advance knowledge on practical applications of human rights-based policies for highly vulnerable people (i.e., refugees, displaced persons, undocumented migrants, and others) during the COVID-19 pandemic in the Global South (Colombia, Ecuador, Peru, Bolivia, and Chile). This project entailed an extensive literature review of scientific and grey literature, analyzing the public policies targeting migrants’ human rights and a qualitative component. For the present manuscript, we will focus on the qualitative component of the Chilean site.

### Context: The Chilean Northern border

Chile’s Northern neighbors are Peru and Bolivia. The Chilean-Peruvian border extends 169 km, and it divides the cities of Tacna in Peru and Arica in Chile. These two border cities share a natural and continuous exchange of goods and services. There is a historical exchange of the Peruvian labor force and the investment and presence of Chilean industry across the border [35]. People may cross more than once in the same day to buy goods, visit their family, and eat.

The Chilean-Bolivian border, over 850 km, divides the Bolivian departments of La Paz, Oruro, y Potosi from the Chilean regions of Arica y Parinacota, Tarapacá y Antofagasta. This region is in the Atacama Desert, one of the world’s most arid regions; therefore, only small cities (e.g., Colchane) with limited resources are found along this border (See Fig. 1).

### Study design

The qualitative component of the larger study considered a case study design. Generally, qualitative methods allow exploring phenomena from an inductive perspective that entails building from particulars and in-depth understanding to general themes, which enables researchers to interpret the collected data and to find patterns within the data eventually. Case study qualitative designs allow in-depth, multi-faceted examinations of complex issues in real-life settings [36]. Therefore, they are helpful when there is a need to obtain an in-depth appreciation of a problem in its natural, real-life context [36]. The purpose of conducting a qualitative case study on this topic in Chile was to deeply describe the case of the health needs and access to healthcare of Venezuelan migrants on the Northern Chilean border during the pandemic from a human rights perspective, as previously defined by the United Nations in 2000 [37] and other similar approaches proposed by international experts [38, 39].

### Participant’s recruitment and data collection

For this case study we conducted two simultaneous data collection techniques: (i) ethnographic participatory observation in the Northern cities of Antofagasta, Iquique, and Arica, close to the northern border, and (ii) semi-structured interviews with key actors. The ethnographic participatory observation aimed to deeply understand the context of migration and human rights at a specific complex point in time. The semi-structured interviews aimed to explore the subjectivity of the context from the participants' point of view [40].

As part of the ethnography conducted for this study, we visited key locations such as migrants’ shelters, local organizations, and places where migrants settled in temporary camps. We held informal conversations with locals to learn about the reality and the overall feeling of how the migrant crisis during the COVID-19 pandemic was dealt with and took detailed ethnographic notes and pictures of essential spots (e.g., plazas, Health Centers) based on a predefined ethnographic data collection chart. Essential public spots were defined based on experts’ recommendations and mass media information around the topic in the country. This chart was designed by the team, piloted in the first visit to the northern area of Chile and adapted accordingly before data collection. We conducted a total of 36 ethnographic observations at public spaces in the three cities. No person was identified nor described in these charts; only a general description of the context and the situation was described in its natural environment.

As for the semi-structured interviews, we created a list of the experts in the three sectors mentioned above and invited them all by email or phone to participate in the in-depth interview, we asked 44 experts to participate, and 30 accepted to participate in our study. We conducted semi-structured interviews with actors of the health sector (*n* = 7), with experts including academics and members of non-governmental organizations (*n* = 16), and with governmental actors (*n* = 7). We purposely chose experts that were part of the co-authors’ social networks, based on over 16 years of research work in this field in the country. We also used the snowball sample technique by asking the participants to provide contacts of some potentially interested peers. Non-probabilistic sampling techniques such as snowball sampling are widely allowed and used in qualitative methods as the aim is to find people willing to share their experiences and that have similar expertise and characteristics as the first invited [41]. Within the large multi-site project, we decided not to interview migrants to avoid revictimization and because we were interested in learning the pre and post COVID-19 pandemic access to health services conditions that were less likely experienced by recently arrived migrants.

Sixteen interviews were conducted via zoom and fourteen in person. Interviews followed a semi-structured guide and included questions on their knowledge of the overall situation at the Northern border during the COVID-19 pandemic, how it may have affected recently arrived migrants, their knowledge of programs focused on migrants, their opinion on the human rights situation, and if they had recommendations to improve migrants’ health access in Chile.

### Data analysis

All in-depth interview audio and ethnographic charts were transcribed verbatim by trained staff, and to protect the participants’ privacy, any personal identifier was removed from the text. Data analyses followed an inductive thematic approach to identify and compare common themes and patterns across participants [40]. The co-authors created a codebook with principal codes and subcodes, guided by human rights-based approaches to health and access to health services during COVID-19 and by the most prominent themes mentioned by the experts interviewed (see methods). The codebook was iteratively revised as data analysis progressed. As a result, we had three different versions of the codebook until the co-authors agreed on a final version [40, 42]. This codebook will be used in the multi-site project to code all interviews conducted in the region.

The available codes were the following: 1) Access to health services based on the Available, Accessible, Acceptable, and of good Quality (AAAQ) Framework, 2) COVID-19 pandemic, 3) International Agreement and Treaties, 4) Human Rights, 5) Social Determinants of Health and Inequities, 6) Receiving Community, 7) Gender perspective, 8) Specific groups and Intersectionality (e.g., gender, children), 9) Interculturality, 10) Public policies and legal instruments, 11) Border and migration processes, 12) Specific diseases and health status and, l3) Recommendations and good practices.

Each general code had sub-codes to provide as much detail as possible to help the authors to conduct a nuanced analysis of each general code. For example, “Specific diseases and health status” included sub-codes such as chronic diseases, dehydration, parasitosis, etc. Given the number of codes and the richness of the data collected, this manuscript focused on codes related to the effects of border closures during the COVID-19 pandemic and how this affected Venezuelan migrants’ human rights arriving in Chile.

The online software Dedoose 9.0.54 [43] was used to manage data coding. Two co-authors (TRJ, CO) coded the in-depth interviews and had weekly meetings to discuss and agree with the coding strategy. As mentioned above, we also analyzed field notes written while visiting the study sites to understand the reality of the human rights of recently arrived migrants, specifically in terms of health and access to healthcare services. All data analysis was conducted in Spanish and after its completion relevant quotes were translated and cross-checked to English by the study team.

## Results

### General description of findings

Overall, we found four dimensions that illustrate the effects of border closure due to the COVID-19 pandemic on Venezuelan migrants’ human rights (i.e., access to health care services) in the Southern Cone region. The first one refers to the precarious conditions that Venezuelan migrants faced while crossing, mainly through irregular crossing points, to Chile. This dimension also explores how the lockdown, and the crisis increased the barriers to accessing healthcare services and the challenges to providing essential services such as shelter and food. The second dimension refers to the new migration patterns and health needs observed during the first months of the COVID-19 pandemic. Most of the participants agreed that the characteristics of the recently arrived migrants were different and diverse from the other flows (e.g., entire families crossing) and their health needs (e.g., chronic diseases). This second dimension also exemplifies the complexity of the migration journey as many of the migrants arriving at this point (2021) passed through other neighboring countries (e.g., Colombia and Peru) and often faced limited access to social and health services in such countries. The third dimension analyzes the barriers to health care, including misinformation, disinformation, discrimination, lack of intercultural competence, and the limited resources faced by the Chilean health care and social system. Irregular entries entailed high barriers to a national unique number (RUT) and other health and social services in the middle of the COVID-19 crisis. The four dimensions explore the regional and local responses in the critical context of the first months of the COVID-19 pandemic. The efforts of the local governments, international organizations, and the local community were vital in assisting the most critical cases of recently arrived migrants. The implementation of local programs that aimed to help migrants living on the streets, such as “Duplas Sanitarias” (Sanitary Pairs), was essential in reaching out to the most vulnerable populations that could not go to the clinic or were afraid to do so. Table 1 summarizes the four dimensions and the content and subthemes of each main theme:

**Table 1:** Dimensions illustrating the impact of border closure due to the COVID-19 pandemic on Venezuelan migrants’ human rights in the Southern Cone region

*#*	*Dimension*	*Sub themes*
1	**Border and migration processes**	• Health issues (e.g., dehydration, accidents including fractures, chronic diseases) while crossing through the Atacama Desert
		• Challenges faced by migrants and locals accessing to basic needs during the most critical point of the pandemic
		• The reality and the consequences of the closure and lock down of shelters and international organizations that aimed to help migrants
2	**Specific groups and Intersectionality**	• Characteristics of the new migrant groups (e.g., entire families) defined as the third Venezuelan wave and their health needs (e.g., mental health issues)
		• Particularities and difficulties of the recently arrived migrants through irregular crossing points that illustrate their complex migration journeys
3	**Barriers to health care services**	• Examples of misinformation, disinformation, and discrimination faced by irregular migrants while trying to access health care services
		• Stateless children and the barriers that the system faced to enter them into the system so they could have access to their fundamental human rights
4	**Regional and local responses to the crisis**	• Description of important efforts taken by international organizations, local organizations, local institutions, community leaders and society to address migrants’ health and social needs
		• Characteristics of two local programs that were implemented to guarantee assistance to basic needs of people living on the streets and to the most vulnerable population (i.e., children and pregnant women)

### Border and migration processes

A recurrent theme in the participants’ testimonies was the precariousness of migrants’ journeys to Chile. Some of the irregular crossing points used are in the Atacama Desert, the most arid region in the world. The COVID-19 pandemic increased the apparent challenges and dangers of crossing through this region in a context in which a high number of migrants were crossing through specific points like Colchane, which are not suitable to receive hundreds every day, and the severe lockdown implemented in Chile. There was very little information on the migration processes during this period of lockdown. Several of the health care providers highlighted the same health issues that they encountered from those who had walked through the dessert:

“We observed issues of dehydration, accidents including fractures, skin burns, malnutrition, chronic diseases like hypertension and diabetes, and dental problems. Crossing through the desert is extremely dangerous” (International NGO, Arica).

The crossing conditions are always complicated in that region; however, the health care system was dealing with the COVID-19 cases therefore, at first, they could do very little to take care of other basic needs. Furthermore, migrants’ primary health needs intersected with structural conditions associated with very limited social networks and the severe lockdown implemented in Chile.

“Migrants had primary care needs due to their migration journey’s they were exposed to the sun and had burns, they didn’t have a place to shower, and they could not go to the local health clinics additionally there were limited shelters or places where they could stay due to the pandemic” (Health care provider, Antofagasta).

As the previous quote illustrates, one of the main consequences for the recently arrived migrants was that they could not access any form of housing, including migrant shelters. Most of the available shelters closed due to the strict measures implemented by the government and the difficulties isolating positive COVID-19 cases. Therefore, the precariousness faced by the recently arrived entailed the migration journey’s problems plus the challenges that the pandemic implicated. Several of the participants mentioned that, especially at the beginning of the pandemic and when borders were recently closed, all was chaos and that there were not enough resources to treat newly arrived migrants nor the local community.

“Approximately there were 500 people crossing every day, it was truly a humanitarian crisis. Migrants would cross through Colchane and then they were put in buses to Iquique only those who were [COVID-19] positive had a place to stay in a sanitary residency during their quarantine. Some of them had to sleep outside and it was very cold at night” (Health care provider, Arica).

It is vital to highlight that the difficulties and challenges faced in this region were not exclusive to the mobile population but also entailed essential concerns to the local population. Border cities like Colchane [border between Chile and Bolivia] were overwhelmed by the presence of hundreds of migrants who were trying to settle in Chile in a context of severe lockdown. The resources were limited overall, and the influx of people into small towns jeopardized the local system's capacity:

“Colchane’s total population is 600 people and suddenly the mobile population added a thousand more and the local health clinic has limited capacity. They only have one doctor, one nurse, one midwife so when migrants and refugees started to arrive, we needed to send health providers to that city, so it did not collapse” (Government, Iquique).

As mentioned earlier, the existing shelters in the Northern cities had to shut down completely as they did not have the infrastructure to isolate suspected or positive COVID-19 cases. Thus, the only mechanism that the government had at the beginning of the pandemic was to test people as they were arriving and those who were positive went to a sanitary residency however, the rest were unassisted, sometimes on the streets or in public spaces. This situation is illustrated by the following quote:

“We had to close the shelter because we did not have the capacity to isolate the COVID-19 cases, so we stopped receiving migrants in March 2020. This meant that people were on the streets with many health needs including trauma” (International NGO coordinator, Arica).

While visiting key locations and conducting ethnographic observations, we saw some people living at the public beaches, on the streets, and on main squares. All the participants that interacted directly with the population shared their concerns of the myriad of health needs that this population was facing in an extreme and complex situation of crossing through irregular points in the middle of a global health emergency.

### Specific groups and intersectionality

Most of the experts who participated in this study agreed that people were crossing regardless of the restrictive measures taken by the governments and that they were observing different migration flows crossing to Chile as well as other health needs in comparison to the previous Venezuelan migrant waves (i.e., 2013–2015; 2015–2018):

“The first wave arrived in 2013 and was characterized by a population with education and wealth that disagreed with the Venezuelan government and could afford to migrate and quickly get a permanent visa. The second wave included academics and professionals who were migrating first by themselves, and then once they were settled, they brought their families and children with them” (NGO member, Antofagasta).

The fact that people were engaging in complicated and dangerous journeys in which there was no certainty whatsoever that they would cross highlights the urgency and desperation of this migrant wave.

“The flow of people entering was steady regardless of border closure, especially the second half of 2020 and we were observing more children, adolescents migrating by themselves, with urgent health needs including pregnant women” (Government, Santiago).

Five participants referred to this wave as the third Venezuelan migrant wave and characterized them as a more precarious group that had overall fewer opportunities in their home country. This highlights social determinants and intersectionality (i.e., ethnicity and social class) that this migrant group experienced before migrating to Chile. Many of these migrants were not coming directly from Venezuela but lived and stayed in other countries such as Colombia and Peru.

“We have observed stories of people who have been out of their country for eight months or a year and were living in other countries like Peru and Ecuador, but the consequences of the pandemic were critical that they could not stay longer in such countries. They could not access basic health and social needs, and they also mentioned experiencing high levels of discrimination” (International NGO coordinator, Santiago de Chile).

The fact that many of the migrants recently arriving to Chile had extensive and complicated migration journey’s that entailed passing and staying in other neighboring countries also illustrates how untenable their living conditions were. Participants also mentioned that in some cases the entire families crossed together including older adults. Therefore, they were dealing with multiple health and social needs at the same time, in comparison to the first migrant waves that brought younger and healthier people:

“This third wave that arrived during and post-pandemic included complete family groups including older adults and sometimes the extended family. They have less education and given the administrative barriers while crossing they also faced authorities’ extortion, they had no savings, and because the entire families were crossing together, they had very little social networks therefore, no place to stay in Chile” (NGO coordinator, Antofagasta).

A vital health issue reported by experts and health providers for older adults was untreated chronic diseases. Many of the recently arrived migrants did not have access to such treatments in their country of origin or in the countries where they spent shorter periods before arriving in Chile:

“Besides the wounds of walking through the desert there were many people with chronic diseases that stopped their treatment for months due to limited access in their home country or other transit countries so when they looked for health care services in Chile they were in bad shape, some of them in critical conditions” (Health care provider, Arica).

An essential and emergent matter was that the most vulnerable populations, such as pregnant women, were not receiving the proper care in their home country or transit locations. Healthcare providers mentioned that there were dozens of women arriving at the very end of their pregnancy in urgent need of prenatal care:

“Many of the pregnant women who we saw crossing through the northern border did not have any prenatal control during their pregnancy they needed a doctor. In some cases, they were taken directly from the border or the sanitary residences to the hospital” (Health care provider, Antofagasta).

Limited access to prenatal care in any country exemplifies urgent issues of fundamental human rights in the region and intersectionality between factors such as gender and migration status. Although the COVID-19 pandemic put all the health and social systems in danger, access to healthcare services of special groups should always be guaranteed. Another group with special needs mentioned in many of the interviews that we did were infants and children; some healthcare providers interviewed said that they also observed infants recently born who were brought through the dessert in hazardous conditions:

“Besides pregnant women, very advanced in their pregnancy, we observed infants in bad shape, sometimes malnourished and we made a huge effort at the health clinic to prioritize the attention of those cases” (NGO coordinator, Arica).

Sometimes infants’ health conditions and health needs were consequences of the conditions in which people were migrating, their migration journeys, and how they entered Chile. Although it is impossible to attend to everyone, special care should always be guaranteed to pregnant women and children regardless of their migration status or form of entry into another country. All the health providers and experts who were interviewed mentioned that they observed health conditions that they do not typically see in Chile and that, at first, they did not know how to attend to or what to do with such cases:

“People arrived with issues that we do not see in Chile like scabies, parasites, sometimes Tuberculosis cases and it took us a while to figure out how to treat some of these conditions. Sometimes migrants would buy antiparasitic drugs for dogs as they could not find it for humans” (Health care provider, Arica).

This context also shows how at the beginning of the pandemic it was hard to keep track of the migrants’ health needs and to incorporate the different practices and social needs into the Chilean health system. Another important health issue that emerged in several interviews was the mental health status of the individuals’ crossing through irregular crossing points during the COVID-19 pandemic. For instance, someone mentioned that the trauma of migrating for the first time in addition to other health issues was critical.

“We received families, with members sometimes old adults that had severe mental health issues as a consequence of the migration journey as well as other unattended chronic health issues” (Public policy expert, Arica).

This matter again intersects with multiple needs that were accumulating during this population’s migration journeys and in which it is likely that they did not have any mental health support to deal with the myriad of traumatic experiences they were dealing with during the entire trip. In addition, other participants mentioned that it was not only the trip but also everything that happened before they began their journey to Chile:

“Migrants and refugees lived unimaginable experiences before they began their journey. They suffered the political instability in their country, the consequences of the pandemic, economic crisis, and then the arrival in Chile” (Community leader, Iquique).

Migrants’ access to mental health services is a critical issue and should be in every country’s agenda. Besides the physical and mental health conditions that migrants had, COVID-19 itself represented a critical issue for the recently arrived individuals as they had no possibility of isolation, many of them were living in crowded environments and arrived to similar contexts once they crossed the dessert. Furthermore, they had very little information and resources to prevent it. A community leader living in a migrant camp in Antofagasta was interviewed and told us at the beginning, they did not know how to deal with those who were infected and they took tremendous efforts to isolate them:

“We did a lot of self-surveillance in the migrant camp, we asked for resources and prevention information to the CESFAM [local clinic] as we did not know what to do…once we identified a case, we helped the family to isolate that person and sometimes we had to inform the local hospital if it was a critical case or needed urgent care” (Community leader, Antofagasta).

This situation intersects with the complications of crossing through irregular points and arriving at a critical pandemic stage. Although the local health system made tremendous efforts to assist the community, limited resources and urgent protocols were missing at the beginning of the pandemic.

### Barriers to health care services

Participants identified a myriad of barriers to health care services that migrants faced. Many of them were exacerbated by the emergency context of COVID-19 but others were associated with the nature of the new migration flows and the migration journeys:

“There was a lot of misinformation and discrimination, sometimes we had cases of health centers not accepting the temporary identification number (NIP) that should be enough for migrants to have access to urgent health conditions such as chronic diseases or pregnancies” (NGO leader, Antofagasta).

Sometimes health providers did not know that migrants without documents could access health care services, and they would return them or give misleading information. Misinformation also included believing someone who had crossed through an irregular border crossing point needed to self-denounce to the local police to access health care services. While conducting visits and ethnographic observations in the bus station, we still saw specific rules applied and implemented, particularly towards migrants who did not have a document that proved their vaccines were up to date. Not everyone knew this rule, so some would lose their bus tickets and the chance to go elsewhere*.* This situation highlights the need for more information on the migration processes and their interaction with the information and the right to access health care.

“The law says that regardless the migration status, everyone has the right to have access to health services including vaccination, especially pregnant women, and children. Sometimes health institutions did not know this or requested a self-denounce proof to provide certain services and some migrants remained unattended. This not only happened at the borders but also in Santiago” (Health expert, Santiago).

Misinformation and disinformation were not only among health providers but also among the recently arrived migrants. They did not know their rights or what they could access in Chile in severe lockdown. This situation was highlighted by several community leaders and non-governmental organization members, as they have observed in previous migrants. The latter knew their rights and sometimes how the Chilean system worked before migrating. But this was something more characteristic of the first waves.

“We observed less educational level and less information overall of the people who were crossing through the Northern border, they did not know that they needed a provisional number (NIP) and even though they were provided with certain flyers or information it was harder to access to any social service as everything was locked down” (NGO Coordinator, Arica).

A critical issue mentioned among a few experts was stateless children and the need to regularize them so they could access basic health services. These children were born in other countries within their migration journey such as Colombia or Peru and they were never registered as they continued their journey to Chile.

“We needed to first register those children and then give them a provisory number so they could access health and social services like education. They were behind in everything doctor’s appointments, education level, so it has been a challenge to provide the basic needs to those children, especially during the closure of the pandemic” (NGO Member, Arica).

Besides registering children and providing a provisory number to those more vulnerable, regularization was a theme mentioned by all our participants as a critical and key factor that needs to be addressed to ensure access to health care services as well as other basic needs such as education.

“We need to solve the issue of regularizing irregular migrants but mainly children, sometimes they do not even have any documentation not even their home country identification which makes it impossible to regularize and to access health and social services” (Public policy expert, Santiago).

Participants’ testimonies emphasize issues to guarantee certain fundamental human rights (e.g., education, access to health care services, access to a nationality) during the first months of the COVID-19 pandemic. However, the emergent themes show that many of the limitations and critical conditions migrants experienced intersect in the South American region. This also elucidates that the responses needed to be regional.

### Regional and local responses to the crisis

Although the situation was critical, mainly at the beginning of the COVID-19 pandemic, with border closures having negative impact on migrants’ access to health services, there were important efforts of international organizations, local organizations, community leaders, and society to address migrants’ health and social needs. We interviewed a few community leaders, and one of them reported being in close contact with the director of the local Health Clinic during the most critical points of the pandemic:

“As a community leader, I felt responsible for all of us who living in the migrant camp. We started asking for help from the director of the CESFAM [local health clinic], and she gave us her phone number so we could ask her about prevention measures, talk to her about the most critical cases, and provide them with surveillance information. Local organizations also helped us with food and other basic products at the most critical point of the lockdown” (Community leader, Antofagasta).

Local health clinics and institutions had to adjust their previous programs to address the needs of migrants crossing through irregular points within the COVID-19 context. With the help of international organizations’ resources, they were able to implement successful programs in the field.

“We observed that we needed to go out to the streets because we could not receive them at the health clinics. We implemented a high level of communication and coordination with other institutions, as the Ministry of Health could not do everything. We designed online courses and followed critical cases by communicating with WhatsApp and other platforms” (Ministry of Health, Arica).

A few members of international organizations mentioned that resources to alleviate the COVID-19 pandemic needs were key to address the humanitarian migrant crisis at the border. A program that was widely mentioned as successful was called “Sanitary Duos” (Duplas Sanitarias), a program that involves international and local government cooperation. This program entails sending a nurse and a technician to assist with the most critical needs in the field. The sanitary duos also reached highly vulnerable populations that were living on the streets, parks, beaches, and squares that were too afraid, due to their migration status, to search for help elsewhere.

“The Sanitary Duos were very key as we reached a level of trust with the official health care services as well as with other institutions. In addition, they entailed interaction with migrants and with local organizations that had to close their doors due to the extreme lockdown” (Ministry of Health, Arica).

“Those Sanitary Duos brought primary care attention to the migrants living on the streets, squares, camps, as they were doing the evaluation, the referrals, they were evaluating their health conditions, and at the same time, they were educating and orienting on the migrants’ human rights” (Public policy expert, Arica).

Another local governmental program that aims to protect children’s health and development widely mentioned in the interviews was “Chile Crece Contigo”. This program was critical for pregnant women as it would follow up with them once the baby was born and would also provide them with other essential resources.

“Most of the migrant pregnant women who were arriving to the CESFAM were in their late pregnancy so we needed to act weekly to process a temporary number so they could enroll in FONASA (public health system) so when they were about to give birth, they already know to which hospital to go and their rights as a mother but also the right of their newborns” (Health care provider, Iquique).

Several participants agreed that more than official programs or institutional initiatives, the collaboration between actors was key to addressing the migrant crisis at the Chilean northern border and that the government, international organizations, non-governmental institutions, and the community worked together to respond to the humanitarian crisis faced in such region. Some of these efforts were focused on social needs such as housing and education for children when they could start going back to school.

“Our focus is on children and their living conditions as one of the basic human rights is to have access to a safe space where they can live and fully develop. We were observing low education levels among recently arrived migrant children, as some of them had been out of school for years. We would help to actualize their knowledge, and with the help of the Ministry of Education, enroll them to public schools and try to ensure stable housing for the kids and their families” (NGO Coordinator, Arica).

During our visits to key locations, we observed how migrant children were participating in activities inside an NGO’s classroom to improve their education level and reach their appropriate school grades.

### Intersections between main study findings: co-occurring codes

As shown in the four previous sections, there are several themes that intersect with each other and that are worth mentioning. The software used to code the in-depth interviews Dedoose, provides with a code co-occurrence analysis. Table 2 shows the most frequent co-occurrent codes, which coincides with what we found in the interviews:

**Table 2:** Coding co-occurrence

Code	Code co-occurrence
Barriers to health care services	State duty in terms of providing information on migration processes and access to health and social services
	Coordination between local, international and regional actors
	No discrimination and equal access to health care
	Governmental response
Border and migration processes	Changes in migration patterns and flows
	Migration status
Specific groups and Intersectionality	Infancy
Coordination between local, international and regional actors	State duty in terms of providing information on migration processes and access to health and social services

Something interesting to highlight from Table 2 is that healthcare accessibility and barriers were multiple times coded with the State’s role of providing information on health and social services. This is also illustrated in the quotes of the section on barriers to health care services in which misinformation and disinformation from providers sometimes increased the obstacles to migrants’ access to health care services. However, as our participants mentioned and the table illustrates, the coordination between local, international, and regional actors was also crucial to ensure healthcare access for the most vulnerable.

Discrimination was also a key factor mentioned by our participants that determined access to healthcare services at the most critical point of the COVID-19 pandemic, and it also appears in Table 2. As our testimonies shared, some of the most critical consequences of border closures are associated with migration patterns changes and migration status. The migration status co-occurrence code is evident as most Venezuelans were crossing through irregular points. Still, something remarkable is that infants are among the groups most affected by the crossing conditions. The role of the State that was not as strong in the testimonies was coded multiple times with other important codes such as the coordination between local, international and regional actors as well as health accessibility.

## Discussion

We qualitatively explored the effects of border closure due to the COVID-19 pandemic on Venezuelan migrants’ human rights, with a focus on access to healthcare, in the Chilean Northern border that connects with Peru and Bolivia. Following a qualitative case study design, we conducted 30 in-depth interviews with experts and 36 ethnographic observations in the three larger cities closest to the border. We found that the main consequences of border closures in the Chilean borders for Venezuelan migrants during the COVID-19 pandemic were: a) Precarious conditions (e.g., dehydration, trauma, structural violence) while crossing through irregular crossing points in the Chilean Northern border with Bolivia and Peru, b) the identification of new migration flows (e.g., pregnant women, older adults, children), and health needs (e.g., chronic diseases, parasitosis, mental health, and trauma) observed during the first months of the COVID-19 pandemic, c) limited access to health care and social services (i.e., misinformation, disinformation, and discrimination due to migration status), and finally, d) urgent and emergent regional and local responses (e.g., Sanitary Duos) in the critical context of the first months of the COVID-19 pandemic.

Our first finding coincides with international research that shows evidence that restrictive border measures negatively affected the physical and mental health of those migrating [17, 19, 20]. A working paper published in 2020 identified that the border restrictions applied in Chile to deal with the Venezuelan migration crisis caused deterioration of people's physical health, including an increased risk of acquiring COVID-19, due to the lack of sanitary control and overcrowding [44]. Studies and reports conducted in other parts of the world during the COVID-19 pandemic found that the restrictive measures imposed affected especially vulnerable groups such as migrants by limiting access to healthcare [45, 46]. An investigation conducted by the U.S. Department of Homeland Security found that COVID-19 negatively affected access to health insurance and healthcare services among irregular migrants [47]. Additionally, a systematic review conducted by Matlin et al. (2022) found that migrants feared that personal data would be transmitted to immigration authorities. Therefore, they did not go to the health care services and were excluded from social protection measures granted by governments [48]. Another paper published by Vera et al. highlights that the vulnerability due to exclusion from social protection affects the population differently and the heterogeneity is determined by the migration status [49]. This situation recognizes the intersectionality across factors such as migration status, ethnicity, and social class that has been widely explored in studies on human rights and access to health care services across vulnerable populations [38, 50].

Our results found a similar pattern in which misinformation, disinformation, and discrimination played an important role in accessing social and health care services among Venezuelan irregular migrants. In February, 2022 the Chilean government published new migratory rules that included a figure of self-denunciation to the Investigations Police of Chile (PDI), this entails that those entering irregularly need to report it to the police to reduce their fine and then follow a series of complex administrative steps to try to avoid deportation [51]. As mentioned in our results section, some health providers thought that migrants needed to self-denounce to access health care services. However, this is not true and since 2015, access to health in Chile was opened to all inhabitants of the country, regardless of migratory status, as stated in the circular A15/06 of the Ministry of Health (2015) [50, 52].

The present study also found that the pandemic affected and changed individual’s migration journeys and trajectories. As the testimonies showed, many of the Venezuelans entering though irregular crossing points were not coming directly from their home country but were living in other neighboring countries such as Peru and Colombia and in the middle of the pandemic they undertook a dangerous trip to enter Chile. This also has been documented in other studies conducted in Chile, in which the pandemic affected migrants’ migratory trajectory as well as deterioration of physical and mental health [50, 52, 53]. It is important to highlight the traumatic experiences that these migrants have probably went through and thus, the unattended psychiatric disorders that they arrived with. The United Nations Refugee Agency 2022 Report of the Northern Chile, found that most of the children and adolescents interviewed experienced emotions such as fear, sadness, anger or have had some kind of difficulty sleeping with varying degrees of intensity [1, 54]. This situation highlights the importance of implementing programs and interventions at border contexts that address trauma and issues in the transit points of the South American region. Although access to mental health services in Chile is limited, recently published studies found that, in general, there are greater tools and increased access to health services than in other countries in the region [53, 55, 56]. In our study, we observe some positive programs and efforts implemented to ensure migrants’ human rights, including access to health services. These efforts were implemented by international organizations, society, and community in coordination with the government showing the importance of communication and solidarity across actors. The aforementioned circumstance aligns with some studies conducted in Chile that have found that the COVID-19 pandemic context was an opportunity to reinforce the perspective that international migrant individuals and communities constitute heterogeneous and dynamic groups in this country [57]. Furthermore, it is essential to highlight that although Venezuelan migrants have faced significant barriers in Chile, it is the Latin American country with the third highest presence of this migrant collective, following Colombia and Peru.

This study has clear implications for public policies and lessons to guarantee migrants’ human rights in the region especially at border contexts. First, we learned from the participants’ testimonies that the coordination between key actors like international organizations, the community, and the local government was crucial to address the humanitarian crisis happening at the beginning of the COVID-19 pandemic [52]. Although there were clear barriers to health care services as well as important human rights issues such as stateless children, there were enormous efforts to address them in the midst of a global crisis. Secondly, the implemented programs in the field such as the Sanitary Duos were also essential to interact with a vulnerable population that were not able to interact with health care providers and that sometimes were afraid of the system [58]. These successful programs should be part of the emergent protocols in the region, and they could even be examples to export to other regions that have similar characteristics to the Northern border of Chile. This study indicates that other humanitarian crises such as climate change, which may produce mass displacements and public health emergencies, should consider the lessons learned during the COVID-19 pandemic. Lastly, this study has shown that restrictive migration policies at borders do not prevent people from crossing through irregular points but do increase the consequences of the precarious crossing conditions and escalates the costs that the receiving country has to cover. In light of the Venezuelan crisis, which has not yet ended, new immigration regulations that respond to the migrant’s human rights need to be implemented.

This study has several limitations. We interviewed 30 experts; therefore, we may not be reflecting all the testimonies and opinions of everyone in the Northern Chilean borders. However, we found critical emergent patterns and themes worth reporting and sharing. One of the strengths of this study is that it includes the experiences of a diversity of actors that may not agree on the diagnosis of the reality during the COVID-19 pandemic. Therefore, we are providing a non-bias analysis of such phenomenon. As our research used qualitative tools, we cannot affirm any association or causality across themes. However, we believe this study provides insight into understanding the effects of border closure due to the COVID-19 pandemic on Venezuelan migrants’ human rights (i.e., access to health care services) in the Southern Cone region.

## Conclusions

In conclusion, the closure of borders due to the COVID-19 pandemic and the ensuing humanitarian crisis showed negative effects in migrant’s human rights, physical, and mental health status. However, local and regional efforts were implemented to address the humanitarian crisis Venezuelans experienced during the most critical period of the pandemic.

## Data Availability

The datasets generated and/or analysed during the current study are not publicly available because they include opinions and perspectives of key members of Chilean governmental institutions and local organizations. We promised privacy and confidentiality but corresponding authors are available upon reasonable request.
